# The role of gut microbiota in patients with benign and malignant brain tumors: a pilot study

**DOI:** 10.1080/21655979.2022.2049959

**Published:** 2022-03-15

**Authors:** Haixiao Jiang, Wei Zeng, Xiaoli Zhang, Yunlong Pei, Hengzhu Zhang, Yuping Li

**Affiliations:** a Department of Neurosurgery, Clinical Medical College of Yangzhou University, Yangzhou, Jiangsu, China; b Department of Clinical Medicine, School of Medicine, Yangzhou University, Yangzhou, Jiangsu, China; c Department of Medical Imaging, The Affiliated Hospital of Yangzhou University, Yangzhou, Jiangsu, China

**Keywords:** Gut microbiota, biomarker, brain-gut axis, tumors, meningioma, glioma

## Abstract

Gut microbiota is associated with the growth of various tumors, including malignant gliomas, through the brain-gut axis. Moreover, the gut microbiota in patients with malignant tumors may considerably differ from those with benign tumors. However, the associations of gut microbiota with benign and malignant brain tumors remain unclear. Hence, in order to explore these underlying relationships, patients with benign meningioma (n = 32), malignant glioma (n = 27), and healthy individuals (n = 41) were selected to participate in this study. The results showed that the diversity of the microbial ecosystem in brain tumor patients were less than the healthy controls, while no significant differences were observed between the meningioma and glioma groups. The microbial composition also differed significantly between individuals with brain tumors and healthy participants. In meningioma group, pathogenic bacteria like *Enterobacteriaceae* were *increased*, whereas certain carcinogenic bacteria were overrepresented in the glioma group, including *Fusobacterium* and *Akkermansia*. Furthermore, benign and malignant brain tumor patients lacked SCFA-producing probiotics. *Thus*, a microbial biomarker panel including *Fusobacterium, Akkermansia, Escherichia/Shigella, Lachnospira, Agathobacter*, and *Bifidobacterium* was established. Diagnostic models confirmed that this panel could distinguish between brain tumor patients and healthy patients. Additionally, gut microbiota can affect the differentiation and proliferation of brain tumors via several metabolic pathways based on annotations from the Kyoto Encyclopedia of Genes and Genomes (KEGG). This is the first study designed to investigate whether gut microbiota differs between benign and malignant brain tumor patients, and our work concluded that intestinal flora is a valuable tool for the diagnosis and treatment of brain tumors.

## Graphical abstract

**Figure:**
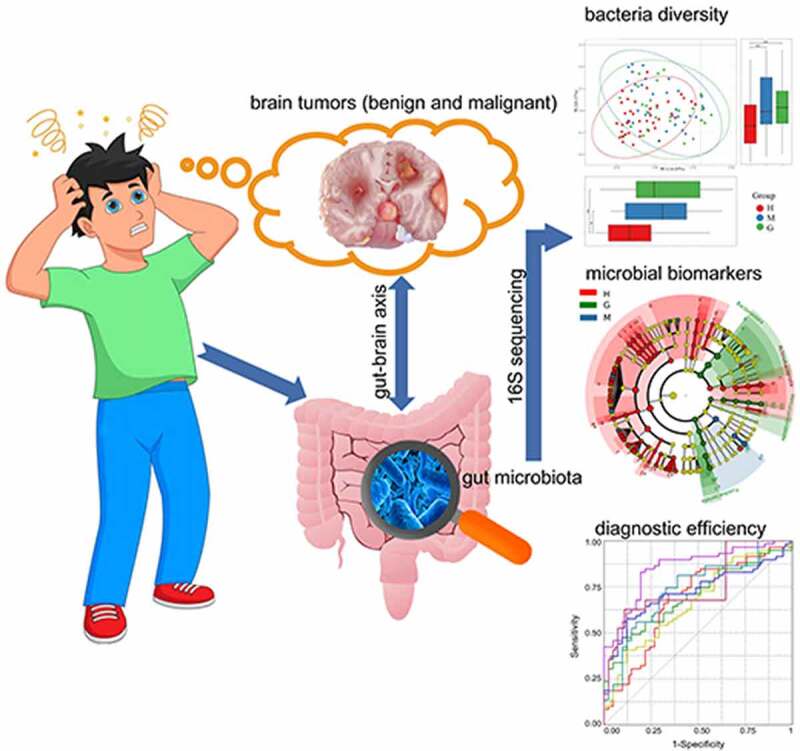

## Introduction

The annual age-adjusted incidence of benign brain tumors is 16.71, while that of malignant ones is 7.08. It is worth mentioning that meningiomas and malignant gliomas are the most common primary brain tumors [1]. Brain tumors can directly affect physiological, emotional, and cognitive functions depending on their location. Moreover, brain tumor patients can incur significant hospital expenses and have a substantially reduced quality of life. Hence, a higher requirement for clinical management is warranted. It is well documented that the best way to improve the prognosis of brain tumor patients is via total tumor resection [2]. However, tumors at advanced stages or malignant tumors could be challenging to excise. Therefore, it would be clinically significant to identify robust biomarkers and effective drug targets for the diagnosis and treatment of brain tumors at an early stage.

It is well-known that the etiology and progression of brain tumors depend on mutation and genetic factors. For example, neurofibromatosis type II (NF2) gene loss can contribute to meningioma formation [3]. Moreover, microRNAs play a significant role in the biological processes of several tumors, and therefore, could be viewed as molecular biomarkers for benign meningioma and malignant glioma [4,5]. However, the molecular biomarkers mentioned above are not widely used in clinical settings for several reasons, such as low detection efficiency and excessive cost. Thus, developing more efficient and low-cost detection methods is vital and meaningful.

The attention given to the relationship between non-genetic endogenous and environmental factors and brain tumors has been increasing [6]. Indeed, with the discovery of the bio-directional pathway termed the gut-brain axis [7], research on the potential role played by gut microbiota in brain tumor patients is becoming ever more important [8]. Gut microbiota is essential for maintaining the intestinal mucosal barrier, immune homeostasis, and metabolic balance [9,10]. It could also be used as a tool to diagnose and treat several tumors [^11–13^]. A potential relationship between intestinal flora and malignant brain tumors has been revealed. Lyu Y et al. and D’Alessandro G et al. observed that by influencing the immune system and modulating neurotransmitters, the crosstalk between intestinal flora and the brain could lead to the formation and development of gliomas [14,15]. This hypothesis was validated by Patrizz A et al. [16] and Xiaochong L et al. [17] in rat experiments, while a human study by Yuqi W [18] proved that oral microbiota is associated with the malignancy of brain tumors. To our knowledge, the composition of intestinal flora is significantly affected by oral microbiota [19]. Furthermore, the gut microbiota in patients with benign and malignant tumors was reported to be distinctly different [20]. Therefore, identifying and comparing diverse intestinal floral structures in malignant and benign brain tumor patients with those of healthy individuals may provide new insights into the noninvasive methods for the diagnosis and treatment of brain tumors.

This is the first human study to investigate the correlation between gut microbiota and benign and malignant brain tumors. We hypothesized that there might be a strong relationship between them, which could be confirmed by 16S rRNA gene sequence analysis [21]. This study was designed to acquire a deeper understanding of the role microorganisms play in the occurrence and development of brain tumors and to identify significant microbial biomarkers as well as possible drug targets for the diagnosis and treatment of brain tumors. Moreover, fecal microbial transplant may become an important therapeutic tool for brain tumors in our future study.

## Materials and methods

### Research design and sample collection

Between June 2020 and June 2021, 60 patients with meningioma (n = 32) and malignant glioma (n = 27) were recruited at the Clinical Medical College of Yangzhou University (Jiangsu, China). To confirm the diagnosis, each patient underwent a detailed medical history evaluation, imaging tests, and postoperative pathological examination. 41 age- and gender-matched healthy controls were recruited. All participants were Jiangsu Han Chinese, and had similar eating habits. Participants were excluded for the following reasons: a family history of gastrointestinal and brain diseases; having undergone medical treatment such as chemoradiotherapy and surgery before sampling; having taken antibiotics or probiotics in the past three months. Each patient was diagnosed with brain tumor for the first time, and their fecal specimens were collected within six hours of hospital admission. The specimens were then placed into a proprietary preservation solution and stored at −80°C. All participants signed the relevant consent forms. This study was registered in the Chinese Clinical Trial Register (ChiCTR2100044256) and approved by the Ethics Committee of the Clinical Medical College of Yangzhou University (2021ky-007-1).

### DNA extraction and 16S rRNA amplicon pyrosequencing

Microbial DNA was extracted from the fecal specimens using a PowerMax (stool/soil) extraction kit (MoBio Laboratories, Carlsbad, USA) [22]. A NanoDrop ND-1000 spectrophotometer (Waltham, USA) was then used to identify DNA quantity and quality. Therefore, quality-checked DNA was obtained and used as a template for amplifying the microbial 16S rRNA gene V4 region, using the forward and reverse primers 515 F (5’-GTGCCAGCMGCCGCGGTAA-3’) and 806 R (5’-GGACTACHVGGGTWTCTAAT-3’). The amplicons were then pooled in equimolar amounts, and the Illumina HiSeq4000 platform was employed for conducting pair-end sequencing.

### Sequencing data analysis

Fast Length Adjustment of SHort reads (FLASH) and Quantitative Insights Into Microbial Ecology (QIIME, v1.9.0) were used to merge and quality-filter all reads [23]. After merging the reads, the singleton or chimera was filtered out. The CD-HIT software (v4.6.1) classified sequences with similarities greater than 97% as an operational taxonomic unit (OTU). A representative sequence was assigned to each OTU, and the Ribosomal Database Project (RDP) Classifier was employed for performing taxon-dependent analysis of individual OTUs. Microbial alpha diversity (α-diversity) was calculated using Shannon, Simpson, and Chao1 indices [24]. For beta diversity (β-diversity), principal coordinate analysis (PCoA) and nonmetric multidimensional scaling (NMDS) were performed in order to evaluate structural alterations in the microbial communities [25,26]. To determine whether the grouping was meaningful, analysis of similarities (ANOSIM) was conducted by comparing inter-group and intra-group differences [27]. The influence of differentially-abundant taxa was evaluated using linear discriminant analysis (LDA) effect size (LEfSe) [28]. Kyoto Encyclopedia of Genes and Genomes (KEGG) was used for abundance and functional annotations. Functional predictions of the metagenome were performed using Phylogenetic Investigation of Communities by Reconstruction of Unobserved States (PICRUSt, v1.1.4) [29]. STAMP software (v2.1.3) was used to further analyze the output file [30]. Finally, the BugBase software was used to measure high-level microbial phenotypes [31].

## Statistical analysis

The R software (v3.2.0) and SPSS (v19.0) were used for data analyses. Differences in continuous variables were calculated using one-way analysis of variance (ANOVA), independent t-test, or Mann-Whitney U-test, whereas categorical variables were calculated using Fisher’s exact test or Pearson chi-square. A *p-*value <0.05 was considered statistically significant.

## Results

More and more animal experiments indicate that gut microbiota may have a significant role in the etiology and progression of gliomas. Based on the fact that gut microbiota could be markedly distinct in benign and malignant tumor patients, we hypothesized that gut microbiota would differ between healthy controls and patients with benign and malignant tumors. Therefore, we aimed to explore the underlying relationships between intestinal flora and brain tumors. 16S rRNA gene sequence analysis revealed profound changes in microbial composition and function of brain tumor patients, particularly in the malignant glioma group. Moreover, a microbial biomarker panel with a favorable diagnostic efficacy was constructed, which could assist in distinguishing brain tumor patients from healthy subjects. According to KEGG (Kyoto Encyclopedia of Genes and Genomes) annotations, gut microbiota may influence brain tumors by several metabolic pathways.

## Participant characteristics

The clinical parameters of all participants are presented in Table 1. No significant demographic differences were observed between the groups. Therefore, no obvious confounding factors affected group discrimination prior to the design of this study.

**Table 1.** The baseline characteristics of all participants

	Meningeoma	Glioma	Healthy controls	HC vs BT	Meningeoma vs Glioma
	(n = 32)	(n = 27)	(n = 41)	*p value*	*p value*
**Age (Mean ± SD)**	55.37 ± 11.47	57.93 ± 9.93	55.80 ± 9.43	0.644	0.361
**Gender**				0.225	0.185
Male	10(31%)	14(52%)	21(51%)		
Female	22(69%)	13(48%)	20(49%)		
**BMI**	23.21 ± 2.33	22.88 ± 2.43	22.36 ± 2.14	0.131	0.595
**Smoking**				0.368	0.657
Absence	30 (93.8%)	26 (96.3%)	37 (90.2%)		
Presence	2 (6.2%)	1 (3.7%)	4 (9.8%)		
**Drinking**				0.600	0.517
Never	27 (84.4%)	22 (81.5%)	35 (85.4%)		
<1 standard drink per day	2 (6.2%)	3 (11.1%)	5 (12.2%)		
≥1 standard drink per day	3 (9.4%)	2 (7.4%)	1 (2.4%)		
**Hypertension**				0.407	0.767
Negative	19 (59.4%)	15 (55.6%)	27 (65.9%)		
Positive	13 (40.6%)	12 (44.4%)	14 (34.1%)		
**Diabetes**				0.211	0.504
Negative	30 (93.8%)	24 (88.9%)	40 (97.6%)		
Positive	2 (6.2%)	3 (11.1%)	1 (2.4%)		
**Hypercholesterolemia**	5 (15.6%)	4 (14.8%)	2 (4.9%)	0.103	0.931
**Excrement regularity**				0.421	0.931
Yes	27 (84.4%)	23 (85.2%)	37 (90.3%)		
No	5 (15.6%)	4 (14.8%)	4 (9.7%)		
**Tumor location**				NA	0.004
Left	15	9	-		
Right	17	10	-		
Diffuse	0	8	-		
**Tumor maximum diameter** **(Mean ± SD, mm)**	40.85 ± 13.45	44.5 ± 14.7	-	NA	0.785
**TNM stage**				NA	<0.0001
Stage I	30 (93.8%)	0 (0%)	-		
Stage II	2 (6.2%)	0 (0%)	-		
Stage III	(0%)	8 (29.6%)	-		
Stage IV	(0%)	19 (70.4%)	-		

HC: Healthy controls. BT: Brain tumors. BMI: Body mass index. SD: Standard deviation. TNM: Tumor-node-metastasis.

## Distribution of gut microbiota in the healthy controls and patients with meningioma and glioma

**Figure 1.**
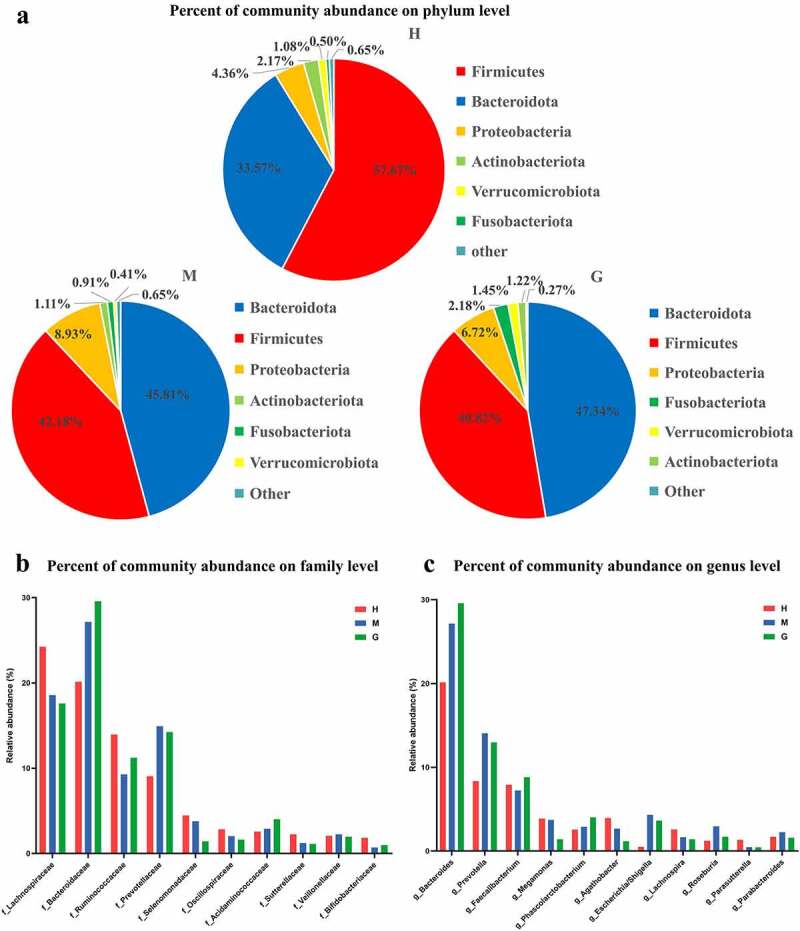
The composition of intestinal flora and the comparison of microbial relative abundance. Pie chart of the dominant phylum-level intestinal flora in healthy subjects and patients with meningioma and glioma (a). The comparison of relative abundance of gut microbiome at the family level (b), and genus level (c) of the three groups. †H: healthy group. M: benign meningioma group. G: malignant glioma group.

A total of 3,761 operational taxonomic units (OTUs) were obtained in this study, including 11 phyla, 63 families, and 149 genera of gut microbes. In order to determine the influence of brain tumors on gut microbiota, microbial composition at different taxonomic levels were established for healthy controls and patients with meningioma and glioma (Figure 1(a-c)).

In the healthy control group, at the phylum level, the most abundant gut microbes were *Firmicutes, Bacteroidetes, Proteobacteria, Actinobacteria, Verrucomicrobia*, and *Fusobacteria* (Figure 1(a)). At the family level, the most abundant gut microbes were *Lachnospiraceae, Bacteroidaceae, Ruminococcaceae*, and *Prevotellaceae* (Figure 1(b)). At the genus level, the most prevalent microbes were *Bacteroides, Prevotella, Faecalibacterium, Agathobacter, Megamonas*, and *Lachnospira* (Figure 1(c)). Similarly, regarding the meningioma group, the most common gut microbes at the phylum level were *Bacteroidetes, Firmicutes, Proteobacteria, Actinobacteria, Fusobacteria*, and *Verrucomicrobiota* (Figure 1(a)). The most common gut microbes at the family level were *Bacteroidaceae, Lachnospiraceae, Prevotellaceae*, and *Ruminococcaceae* (Figure 1(b)). The most abundant gut microbes at the genus level were *Bacteroides, Prevotella, Faecalibacterium, Escherichia/Shigella, Megamonas*, and *Roseburia* (Figure 1(c)). As for the glioma group, at the phylum level, the most common microbes were *Bacteroidetes, Firmicutes, Proteobacteria, Fusobacteria, Verrucomicrobiota*, and *Actinobacteria* (Figure 1(a)). The most common microbes at the family level were *Bacteroidaceae, Lachnospiraceae, Prevotellaceae*, and *Ruminococcaceae* (Figure 1(b)). The most prevalent gut microbes at the genus level were *Bacteroides, Prevotella, Faecalibacterium, Phascolarctobacterium*, and *Escherichia/Shigella* (Figure 1(c)). The relative abundances of these bacteria are outlined in Supplementary Table S1.

## Microbial diversity in patients with meningioma and glioma

**Figure 2.**
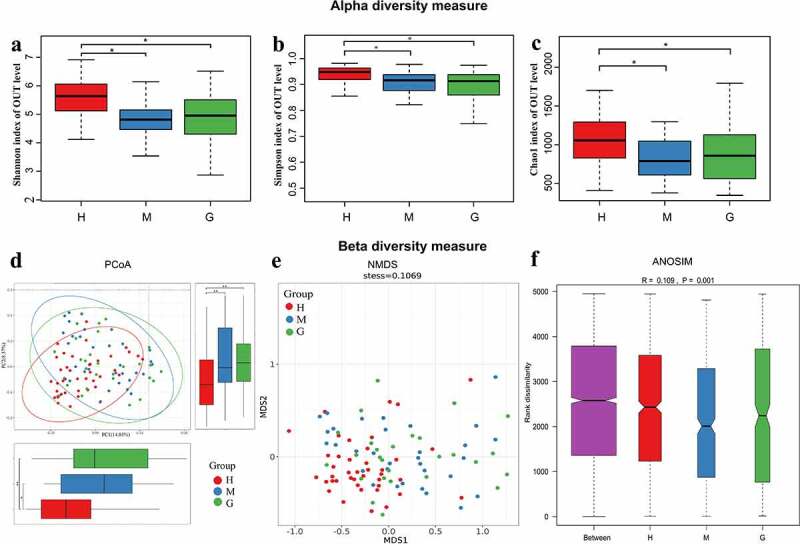
The alpha and beta diversity of the intestinal flora in healthy subjects and patients with meningioma and glioma. Boxplot of alpha diversity indices, including Shannon (a), Simpson (b), and Chao1 (c) indices. Principal coordinate analysis (PCoA) plot based on unweighted UniFrac distances (A). The horizontal and vertical axes represent the first and second principal coordinates, which explains the greatest proportion of variance to the microbial communities. Each spot represents a sample, and each color represents a group. * 0.01 < *p* < 0.05; ** *p* < 0.01; Non-metric Multidimensional Scaling (NMDS) based on Bray-Curtis distances (B). Each spot represents one sample, and each color represents one group. The samples with high similarity of gut microbiome compositions present a close distance in spots. The analysis of similarities (ANOSIM) in healthy subjects and patients with meningioma and glioma (C). The result shows the differences in the inter-group to be significantly greater than in the intra-group (R = 0.109, *p* = 0.001). †H: healthy group. M: benign meningioma group. G: malignant glioma group.

The α-diversity indices (Shannon, Simpson, and Chao1) were all found to be significantly reduced in brain tumor patients, compared to the healthy controls, while differences between meningioma and glioma groups were not apparent (Figure 2(a-c)). Furthermore, both principal coordinates analysis (PCoA) and non-metric multi-dimensional scaling (NMDS) analysis suggested that the intestinal flora of brain tumor patients clusters differently compared to that of healthy controls (Figure 2(d-e)). In addition, the analysis of similarities (ANOSIM) signaled that the microbial structure of each group was significantly different (Figure 2(f)).

## Potential microbiota biomarkers of brain tumors

**Figure 3.**
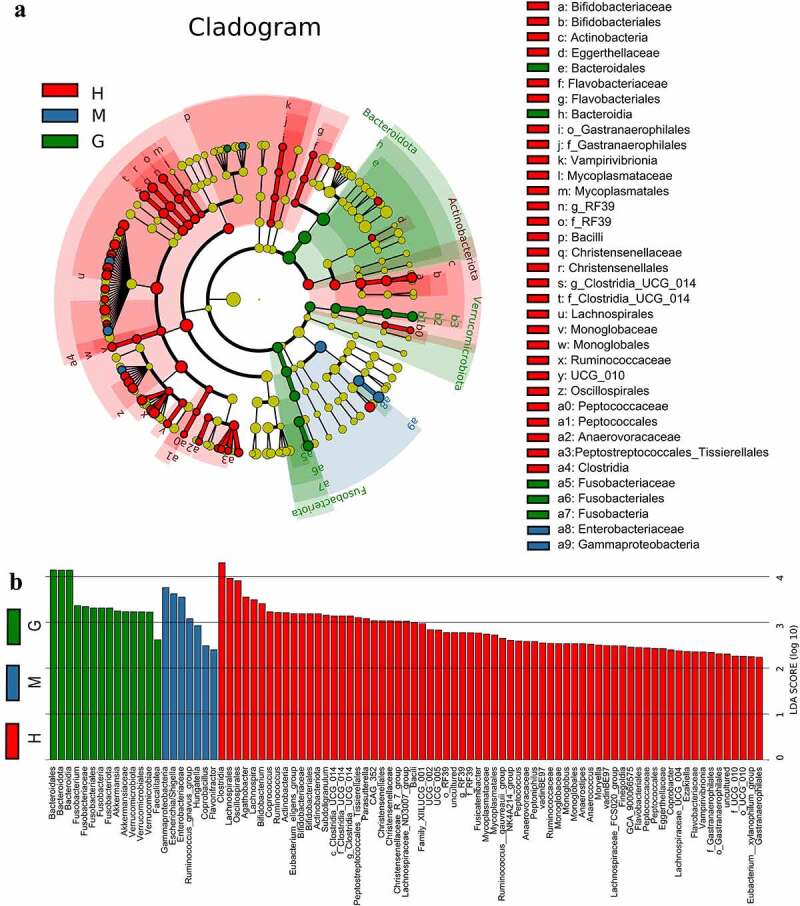
Linear discriminant analysis (LDA) coupled with effect size (LEfSe). Cladogram suggests enriched taxa in intestinal flora of the healthy subjects and patients with meningioma and glioma (a). The LDA scores of different microbial taxa in healthy subjects and patients with meningioma and glioma (b). The radiations from inside to outside indicate the taxonomic levels from phylum to genus. One node indicates one species classification at that level. The yellow node suggests that the species was not different between groups, and nodes in other colors suggest that the species was enriched in the corresponding group. Only taxa with an LDA value greater than 2 are displayed. †H: healthy group. M: benign meningioma group. G: malignant glioma group.

**Figure 4.**
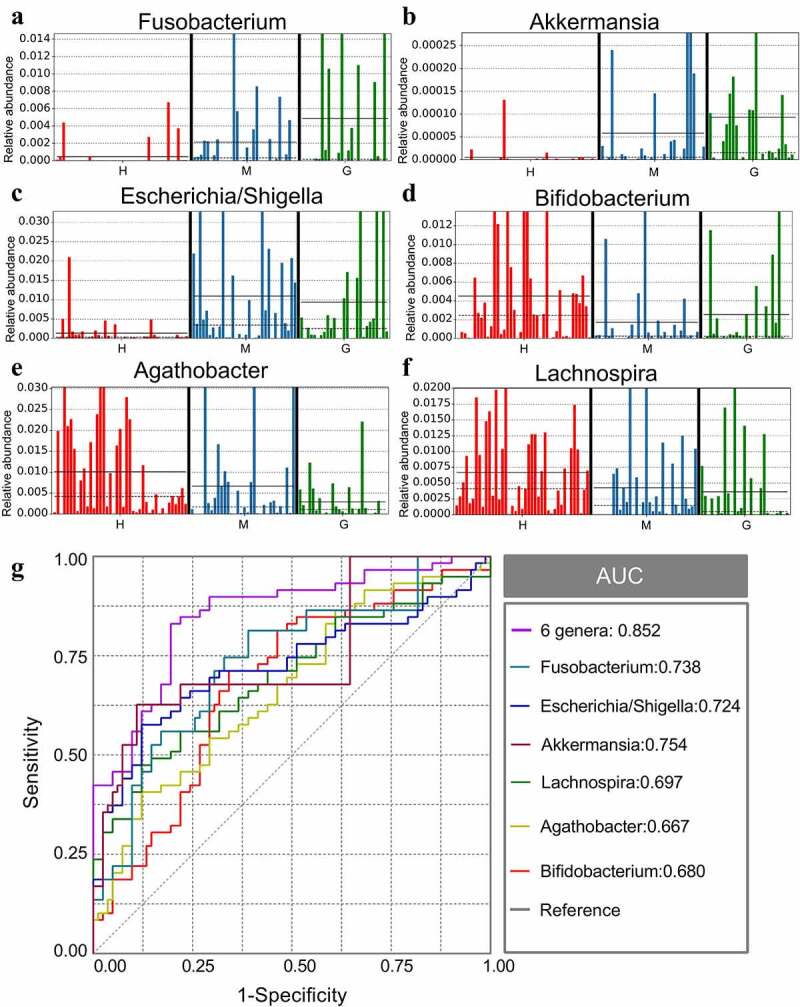
The different genera as biomarkers for brain tumors. The relative abundance of different genera, including Fusobacterium (a), Akkermansia (b), Escherichia/Shigella (c), Lachnospira (d), Agathobacter (e), and Bifidobacterium (f) per sample. ROC curves were calculated to distinguish patients with brain tumors and patients in the healthy group according to the six genera, either individually or combined (g). †ROC: receiver operating characteristic; AUC: area under the receiver operating characteristic curve. H: healthy group. M: benign meningioma group. G: malignant glioma group. c: class; o: order; f: family; g: genus.

Gut microbiota composition was analyzed at all taxonomic levels with LEfSe, and 89 brain tumor-associated microbial taxa were identified (7 meningioma-enriched, 14 glioma-enriched, and 68 healthy-enriched) (Figure 3(a-b)). Several pathogenic and carcinogenic bacteria were discovered in brain tumors, particularly in the glioma group, while numerous probiotics were overrepresented in the healthy controls. Six abundant genera, namely *Fusobacterium, Akkermansia, Escherichia/Shigella, Lachnospira, Agathobacter*, and *Bifidobacterium*, were solely and jointly assessed as biomarkers of brain tumors. The significant inter-personal variations of these genera are delineated in Figures 4(a-f). In addition, ROC curves were graphed, and the results revealed that the combination of these genera significantly increased predictive performance (Figure 4(g), AUC:0.852). Collectively, these findings established that microbial panel is closely associated with brain tumors and that this diagnostic model is appropriate.

## Gene functional prediction

**Figure 5.**
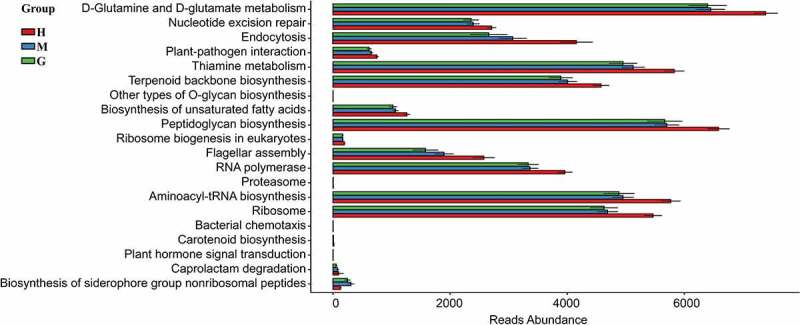
Functional predictions in the intestinal flora of healthy subjects and patients with meningioma and glioma. The top 20 significantly different KEGG pathways among the groups were determined using PICRUSt (*p* < 0.05). †KEGG: Kyoto Encyclopedia of Genes and Genomes. PICRUSt: Phylogenetic Investigation of Communities by Reconstruction of Unobserved States. H: healthy group. M: benign meningioma group. G: malignant glioma group.

PICRUSt analysis was conducted to predict the gut microbial functions of brain tumor patients. Consequently, 137 KEGG modules were obtained, and the top 20 different KEGG pathways among the three groups can be visualized in Figure 5. Based on annotations from the KEGG database, 17 pathways of the brain tumor patients were found to be significantly decreased, including the metabolism of D-Glutamine and D-glutamate, the repair of nucleotide excision, and endocytosis. At the same time, the pathways for the glioma group showed a greater tendency to decrease compared to the meningioma group. Therefore, the dysfunction of the gut microbiome may contribute to the growth of brain tumors and is correlated with the risk of malignancy.

## Discussion

Recently, it was reported that gut microbiota is associated with various tumors and neurological disorders affecting immune and neurological functions [32,33]. Moreover, the gut microbiota could be a useful tool for the diagnosis and treatment of diseases [34,35]. In addition, the function of the gut microbiota in the glioma-bearing mice model has been determined, and the intestinal flora may vary considerably between patients with benign and malignant tumors. All the evidence implies a strong relationship between intestinal flora and brain tumors. However, the exact mechanisms and correlations remain unclear. Hence, this is the first human study designed to explore gut microbiota in brain tumor patients by 16S rRNA amplicon pyrosequencing. Previous animal studies mostly focused on malignant gliomas. To obtain a more credible conclusion, both benign and malignant tumors were considered. Furthermore, a microbial biomarker panel was constructed for the early detection of brain tumors, which is a promising field for clinical application [36]. We also predicted the gut microbial functions of patients with brain tumors for the first time. These could provide a better understanding of the relationship between gut microbiota and brain tumors and present a new perspective on biomarkers and possible drug targets for the diagnosis and treatment of brain tumors.

Profound microbial structural changes were discovered in brain tumor patients. These are concordant with the outcomes of previous mice experiments [16,37]. At the phylum level, the *Firmicutes* and *Bacteroidetes* were the dominant flora of each group, while the Firmicutes to Bacteroidetes ratio (F/B) declined in brain tumors. Low F/B was found to decrease circulating short-chain fatty acids (SCFAs, mainly comprised of butyrate, propionic acid, and acetic acid) and induce metabolic dysfunction [38]. Changes at the family and genus levels were more complex. The three decreased α-diversity indices implied that the richness and evenness of microbial ecosystem in brain tumor patients were significantly lower than the healthy controls. It is widely known that the loss of the microbial ecosystem could contribute to chronic diseases [39]. All the evidence suggests that gut microbiota equilibrium in patients with brain tumors was disrupted. We postulated that the disturbed central nervous system could trigger the disturbed gut microenvironment through the ‘brain-gut axis,’ which needs to be confirmed in future animal studies. Furthermore, no significant difference was discovered in microbial diversity between meningioma and glioma patients. We postulate that a larger sample size will be required to explore the underlying differences.

The underlying brain tumor-associated gut microbiota was revealed in this study. The typical pathogen *Enterobacteriaceae* was overrepresented in meningioma [40] and could also suppress SCFA-producing bacteria and contribute to the dysbiosis of immune and intestinal environments [41]. The genus *Escherichia/Shigella* has been reported in studies of brain tumors and can promote chronic neurological inflammation by neurotoxicity [42]. There were five other meningioma-enriched pathogens, suggesting an imbalanced gut microbial environment in benign brain tumors. Moreover, 14 glioma-associated bacteria (including carcinogenic pathogens) were detected, including *Fusobacterium* and *Akkermansia* [43]. The genus *Fusobacterium* can stimulate the development of malignant tumors by mediating the expression and activity of DNA methyltransferase [44,45]. On the other hand, the *Akkermansia* genus can induce inflammatory responses, neurotoxicity, and blood-brain barrier disruption in the microenvironment of gliomas through its ability to degrade the intestinal mucosal layer [16]. Therefore, glioma patients were found to have a worse dysbiosis in gut microbiota, indicating that gut microbiome signatures are relevant to the risk of malignancy. In addition to exploring the underlying pathogens, this study also evaluated the low-abundance bacteria in brain tumor patients, including *Lachnospira, Agathobacter*, and *Bifidobacterium*. Both the *Lachnospira* and *Agathobacter* genera may possess anti-tumor activity through butyric acid production [46,47]. Butyrate is mainly generated by anaerobic bacteria and plays an essential role in gut physiology [48]. Moreover, butyrate is associated with the intestinal cell life cycle and can prevent the invasion of pathogens, slow tumor progression, and modulate immune responses in CNS [49]. In addition, the genus *Bifidobacterium* belongs to the probiotic family and is instrumental in the dynamic balance of immunity, neurohormone, and metabolism [50]. Therefore, reducing beneficial bacteria may be a risk factor for brain tumors. Moreover, a diagnostic model for brain tumors was established using six abundant bacteria, and the AUC area exhibited satisfactory predictive performance. All the evidence advocates for intestinal flora to be used as a diagnostic tool.

Furthermore, the most notable change in the KEGG pathways of brain tumors (particularly in malignant glioma) was the reduction in the metabolism of D-Glutamine and D-glutamate. They can translate each other and participate in the tricarboxylic acid (TCA) cycle, the dysbiosis of which is associated with the pathogenesis and development of tumors [51]. Additionally, the decreased pathways in nucleotide excision repair (NER) and endocytosis in brain tumors insinuate a disruption in epigenetics and cellular processes [52,53]. NER is a key DNA repair mechanism that can remove helices distorting DNA adducts. NER defects are linked to neurodegeneration and tumors by triggering unrepaired lesions, including apoptosis and enforcing cell elimination [54]. To summarize, the interactions between brain tumors and intestinal flora are complex and not fully understood. Besides, disturbances in the microbiome-metabolome interface may result in the formation and development of various brain tumors. It can be hypothesized that this process may be induced by tumor-promoting metabolites and toxins involved in inflammatory and immune responses. Therefore, specific pathways can be used as diagnostic and therapeutic targets for brain tumors.

This study is the first to reveal the remarkable microbial structural and functional changes in brain tumor patients, including the reduction of SCFA-producing bacteria (such as *Lachnospira, Agathobacter*, and *Bifidobacterium*), the increase in pathogenic and carcinogenic bacteria, and the dysbiosis of metabolic pathways. Meanwhile, the varying degrees of microbiota dysfunction are associated with the risk of malignancy in brain tumor patients. All the evidence from this pilot study points out that gut microbiota plays a vital role in the diagnosis and treatment of brain tumors, which could be confirmed in future studies. Furthermore, animal models should be established to explore additional adjunctive therapeutic tools for brain tumors, including modulating gut microbiota by specific drugs and even fecal microbial transplant.

There are some limitations to this study. Firstly, the cross-sectional study design lacks causal effectiveness and a longitudinal view of relevance. Secondly, only patients with meningioma and glioma were enrolled, as these are the most prevalent benign and malignant brain tumors, respectively. Enrolling more subtypes and samples is necessary to reach more credible conclusions. Thirdly, it is impractical to eliminate all the factors of bias correlating with intestinal flora. Another major limitation of this study is the lack of animal experiments testing mechanisms. Hence, future experiments should be designed more elaborately while addressing these issues.

## Conclusion

In conclusion, significant dysbiosis in the structure and function of gut microbiota was observed in brain tumor patients, particularly in those from the malignant glioma group. Moreover, a microbial panel of *Fusobacterium, Akkermansia, Escherichia/Shigella, Lachnospira, Agathobacter*, and *Bifidobacterium* could be used as biomarkers for brain tumor patients. Seemingly, the effects of intestinal flora on brain tumors were noted in inflammatory and immune pathways. This pilot experiment preliminarily suggests that gut microbiota could be a promising tool for the diagnosis and treatment of brain tumors. However, animal studies and larger prospective cohort human studies are warranted to validate the results of our study.

## Supplementary Material

Supplemental Material
