# Supplementary material for: The role of gut microbiota in patients with benign and malignant brain tumors: a pilot study
Source: Bioengineered. 2022 Mar 15;13(3):7846–58. doi: 10.1080/21655979.2022.2049959 (PMC9208447; doi:10.1080/21655979.2022.2049959)
Supplement: Supplemental Material [file KBIE_A_2049959_SM5665.docx]

**Supplementary Table 1. The relative abundance of** **[dominan](javascript:;)t gut microbiota among** [**meningioma**](about:blank)**, glioma and healthy control groups.**

|  | mean_H (n=41) | mean_M (n=32) | mean_G (n=27) |
| --- | --- | --- | --- |
| p_Firmicutes | 0.576729512 | 0.421765625 | 0.408151852 |
| p_Bacteroipota | 0.335715122 | 0.458093125 | 0.473403333 |
| p_Proteobacteria | 0.043635366 | 0.089314063 | 0.067183704 |
| p_Actinobacteriota | 0.02166122 | 0.011108125 | 0.012195926 |
| p_Verrucomicrobiota | 0.010819024 | 0.004059688 | 0.014465556 |
| p_Fusobacteriota | 0.005 | 0.0090575 | 0.021779259 |
| p_pesulfobacterota | 0.004577561 | 0.003962188 | 0.002651111 |
| p_Campilobacterota | 0.001061463 | 0.002186875 | 2.2963E-05 |
| f_Lachnospiraceae | 0.242349756 | 0.185787188 | 0.175819259 |
| f_Bacteroidaceae | 0.201387073 | 0.271494688 | 0.295807407 |
| f_Ruminococcaceae | 0.139519268 | 0.092809688 | 0.112206296 |
| f_Prevotellaceae | 0.090632683 | 0.1492125 | 0.142474074 |
| f_Selenomonadaceae | 0.044588049 | 0.037648438 | 0.014257778 |
| f_Oscillospiraceae | 0.028337317 | 0.020183125 | 0.016345185 |
| f_Acidaminococcaceae | 0.025723902 | 0.02898125 | 0.040177037 |
| f_Sutterellaceae | 0.022397805 | 0.012225938 | 0.011145926 |
| f_Veillonellaceae | 0.020691951 | 0.022392813 | 0.019696667 |
| f_Bifidobacteriaceae | 0.018315366 | 0.006994688 | 0.009926667 |
| g_Bacteroides | 0.201387073 | 0.271494688 | 0.295807407 |
| g_Prevotella | 0.083575122 | 0.140595 | 0.129741111 |
| g_Faecalibacterium | 0.079217561 | 0.07237 | 0.08829037 |
| g_Megamonas | 0.038733415 | 0.037109688 | 0.014125556 |
| g_Phascolarctobacterium | 0.025496585 | 0.028964063 | 0.040074074 |
| g_Agathobacter | 0.039237073 | 0.026777813 | 0.011693704 |
| g_Escherichia/Shigella | 0.005010244 | 0.043048125 | 0.036258148 |
| g_Lachnospira | 0.025727805 | 0.016402813 | 0.014032222 |
| g_Roseburia | 0.012300488 | 0.029480938 | 0.016996667 |
| g_Parasutterella | 0.013212683 | 0.004609688 | 0.004431111 |
| g_Parabacteroides | 0.016992195 | 0.022547188 | 0.015917407 |

Abbreviations: HC, healthy control; M, [meningioma](about:blank); G, glioma; p, phylum; f, family; g, genus.
